# Dietary Inflammatory Index and Mortality from All Causes, Cardiovascular Disease, and Cancer: A Prospective Study

**DOI:** 10.3390/cancers14194609

**Published:** 2022-09-22

**Authors:** Zhen Liang, Yanfei Feng, Nitin Shivappa, James R. Hebert, Xin Xu

**Affiliations:** 1 Department of Urology, First Affiliated Hospital, School of Medicine, Zhejiang University, Hangzhou 310003, China; 2 The Second Clinical Medical College, Zhejiang Chinese Medical University, Hangzhou 310053, China; 3 Cancer Prevention and Control Program, University of South Carolina, Columbia, SC 29208, USA; 4 Department of Epidemiology and Biostatistics, Arnold School of Public Health, University of South Carolina, Columbia, SC 29208, USA; 5 Department of Nutrition, Connecting Health Innovations LLC, Columbia, SC 29201, USA

**Keywords:** dietary inflammatory index, DII, mortality, prospective, PLCO

## Abstract

**Simple Summary:**

Our study investigated the association between the inflammatory potential of the diet, as calculated by the Energy-adjusted Dietary Inflammatory Index (E-DII^TM^), and total and cause-specific mortality. We analyzed the data of 101,832 participants from the Prostate, Lung, Colorectal and Ovarian (PLCO) trial with 17 years of follow-up. We found that the E-DII was significantly associated with all-cause mortality. The HR (95% CI) in the highest E-DII quintile compared to the lowest quintile was 1.23 (1.18–1.29) (*p* for trend <0.001). Based on the fully adjusted model, the E-DII was also statistically significantly associated with CVD and cancer mortality. The findings of our study provide more evidence to help guide recommendations regarding anti-inflammatory food intake.

**Abstract:**

The Energy-adjusted Dietary Inflammatory Index (E-DII^TM^) is a comprehensive, literature-derived index for assessing the effect of dietary constituents on inflammatory biomarkers and inflammation-related chronic diseases. Several studies have examined the association between E-DII scores and mortality, with results that vary across populations. Therefore, in the present study, we aimed to investigate the potential association between E-DII scores and all-cause, cardiovascular disease (CVD), and cancer mortality using data from the Prostate, Lung, Colorectal and Ovarian (PLCO) Screening Trial. E-DII scores, calculated based on a food-frequency questionnaire, were analyzed both as a continuous variable and after categorization into quintiles. A multivariate Cox proportional hazards model was used to estimate hazard ratios (HRs) and 95% confidence intervals (CIs). A total of 101,832 individuals were included, with 24,141 deaths recorded after a median of 17.0 years of follow-up. In multivariable-adjusted analyses, the E-DII score was significantly associated with all-cause mortality. The HR (95% CI) in the highest E-DII quintile compared to the lowest quintile was 1.23 (1.18–1.29). The E-DII was also statistically related to CVD mortality (Q5 vs. Q1; HR, 1.30 [95% CI, 1.20–1.41]) and cancer mortality (Q5 vs. Q1; HR, 1.14 [95% CI, 1.06–1.24]). Similar results were obtained from sensitivity analyses and subgroup analyses. In conclusion, the inflammatory potential of the diet, as calculated by the E-DII, was significantly associated with overall and CVD- and cancer-specific mortality risk in the PLCO study.

## 1. Introduction

Chronic inflammation represents a long-term pathological condition characterized by a continued active inflammation response and tissue destruction [1]. Many studies have found that chronic inflammation is related to a wide variety of human diseases, including diabetes, cardiovascular diseases (CVDs), autoimmune diseases, and cancer [1,2]. It has been proposed that dietary strategies can modulate inflammatory activity [3,4]. Several important bioactive dietary components can interfere with selective inflammatory pathways to affect metabolic and genetic changes [3]. The whole-diet approach seems particularly promising for reducing the levels of inflammation compared with individual foods or food constituents [5,6].

The Dietary Inflammatory Index (DII^®^) was developed to assess the inflammatory potential of the diet [7]. This index is based upon an extensive literature search incorporating cell culture, animal, and human studies on the potential effects of the diet on inflammation [8]. A higher DII score indicates a more pro-inflammatory diet, while a lower value represents a more anti-inflammatory diet. Nearly 90 studies have used the DII to evaluate the association between dietary inflammatory potential and all-cause and cause-specific mortality with inconsistent results. Veronese et al. [9] reported that higher DII scores were associated with a significantly higher total mortality risk, whereas no associations were found between DII scores and cancer or cardiovascular disease (CVD) death based on a cohort longitudinal study in a Mediterranean area. Okada et al. [10] found that the DII was significantly associated with all-cause and CVD mortality but not with cancer mortality in middle-aged and older Japanese adults. The findings from the SUN cohort and PREDIMED trial and a meta-analysis of 12 prospective studies indicated that a pro-inflammatory diet, as estimated by the DII, was significantly associated with increased all-cause mortality [11]. Overall, evidence for the DII in relation to all-cause mortality has generally been strong and consistent, whereas the associations of the DII with cause-specific mortality, especially cancer mortality, have been less clear. The objective of this study was to further assess the association between the DII and the mortality risk using data from the Prostate, Lung, Colorectal and Ovarian (PLCO) study, a large screening trial conducted by the US National Cancer Institute.

## 2. Materials and Methods

### 2.1. Study Population

The PLCO study was a multi-center population-based randomized trial designed to assess whether certain screening tests reduce death from prostate, lung, colorectal, and ovarian cancer [12]. The specific introduction of the PLCO study design was stated in our previous papers [13,14]. The approved number of this study is PLCO-587.

### 2.2. Cohort Selection and Criteria for Exclusion

Participants were excluded from this study if they did not return a baseline questionnaire (BQ) (n = 4918); had cancer diagnosed before completing the diet history questionnaire (DHQ) (n = 10,199); did not have follow-up data (n = 12); or did not complete the DHQ or the DHQ was not valid (n = 37,936). After these exclusions, the analytical cohort included a total of 101,832 subjects.

### 2.3. Data Collection 

The individuals completed a BQ containing baseline information such as demographic characteristics and lifestyle factors. Dietary data were collected using the DHQ version 1.0 (National Institutes of Health, Applied Research Program, National Cancer Institute. 2007), which recorded the frequency of consumption and portion size of 124 food items and supplement use over the past year [15]. The DHQ has been validated against four 24 h dietary recalls among 1640 nationally representative participants in the Eating at America’s Table Study [15]. Daily nutrient intake was calculated by the DietCalc software, which integrated responses of food frequency, portion size, and other responses using national dietary data from adults in USDA’s Continuing Survey of Food Intake by Individuals 1994–1996 and supplemented by the Nutrition Data Systems for Research (NDS-R) from the University of Minnesota [16].

### 2.4. Energy-Adjusted DII (E-DII) Score Calculation

The DII is a literature-derived, population-based dietary index developed as a comprehensive index to evaluate the overall inflammatory potential of an individual’s diet. Details regarding the DII are described elsewhere [7]. The DII has been construct-validated in 30 studies and consistently found to be associated with higher levels of inflammatory biomarkers, including IL-6 [17], TNF-α receptor 2 [17], and high-sensitivity CRP [18]. The DII consists of 45 dietary factors. The DHQ in the present study provided data on 35 food parameters as listed below: Alcohol, Vitamin B12, Vitamin B6, β-Carotene, Caffeine, Carbohydrates, Cholesterol, Energy, Total fat, Fiber, Folic acid, Fe, Mg, MUFA, Niacin, Onion, Protein, PUFA, Riboflavin, Saturated fat, Se, Thiamin, Trans fat, Vitamin A, Vitamin C, Vitamin D, Vitamin E, Zn, Green/black tea, Flavan-3-ol, Flavones, Flavonones, Anthocyanidins, Isoflavones, and Pepper.

The specific formula is: Z-score = (individual reported intake-global daily mean intake)/global standard deviation. To minimize right skewing, the Z-score was converted to a centered percentile score [(2* percentile of Z value −1)]. Finally, the result was multiplied by the total inflammatory score of each dietary component, and the results were combined to obtain the personal DII score. We calculated E-DII scores based on intake standardized to 1000 kcal of energy [8]. A higher E-DII score indicates a more pro-inflammatory diet, while a lower value represents a more anti-inflammatory pattern.

### 2.5. Outcome Assessment

All study participants were followed from the date of DHQ completion to the time of death or through 2015. The specific outcome assessment method was stated in our previous papers [13,14]. The primary outcomes of interest were all-cause mortality and mortality from CVD or cancer.

### 2.6. Statistical Analysis

The E-DII score was analyzed both as a continuous variable and after categorization into quintiles (Q). E-DII scores ranged from the negative tail to the positive tail; Q1 had the highest anti-inflammatory properties, while Q5 had the highest pro-inflammatory properties. A multivariate Cox proportional hazards model was used to estimate hazard ratios (HRs) and 95% confidence intervals (CIs). The model was adjusted for various potential confounders, including age (continuous), sex (categorical), randomization arm (categorical), race (categorical), body mass index (BMI, continuous), education (categorical), marital status (categorical), smoking status (categorical), aspirin use (categorical), history of hypertension (categorical), history of diabetes (categorical), history of stroke (categorical), and history of heart attack (categorical).

Subgroup analyses were performed based on age, arm, sex, smoking status, BMI, education level, and race. Sensitivity analyses were performed by excluding deaths that occurred within 2 or 5 years of follow-up. Interaction assessment analysis and dose–response analysis were described in our previous papers [13,14]. All statistical analyses were performed using the software STATA version 15 (Stata Corp, College Station, TX, USA) with two-sided *p*-values.

## 3. Results

### 3.1. Study Characteristics

Our study included a total of 101,832 individuals and recorded 24,141 deaths after a median of 17.0 years of follow-up. These deaths included 7534 from CVD, 7161 from cancer, and 9446 from all other causes combined. The average age of participants at baseline was 62.4 (SD 5.3) years. The median E-DII was −4.0 (−8.6 to 5.8). The participants with higher E-DII scores were more likely to be female, be current smokers, be married, have a lower level of education, and have a higher BMI. The main characteristics are summarized in Table 1.

### 3.2. E-DII and All-Cause Mortality

As shown in Table 2, in multivariable-adjusted analyses, the E-DII, fit as quintiles, was significantly associated with all-cause mortality. The HR (95% CI) in the highest E-DII quintile compared to the lowest quintile was 1.23 (1.18–1.29), *p* for trend <0.001. When the E-DII was analyzed as a continuous variable, the HR (95% CI) of one-unit increment in the E-DII for all-cause mortality was 1.05 (1.04–1.06).

### 3.3. E-DII and Cause-Specific Mortality

Based on the fully adjusted model, the E-DII was statistically significantly associated with CVD mortality (Q5 vs. Q1; HR, 1.30 [95% CI, 1.20–1.41], *p* for trend <0.001) (Table 2). In the continuous analysis, the adjusted HR was 1.06 (95% CI 1.04–1.08) per one-unit increment of the E-DII. An increased E-DII was also significantly associated with higher cancer mortality (Q5 vs. Q1; HR, 1.14 [95% CI, 1.06–1.24], *p* for trend = 0.001), and HR was 1.03 (95% CI 1.01–1.05) per one-unit increment of the E-DII when fit as a continuous variable.

### 3.4. Additional Analyses

**Figure 1:**
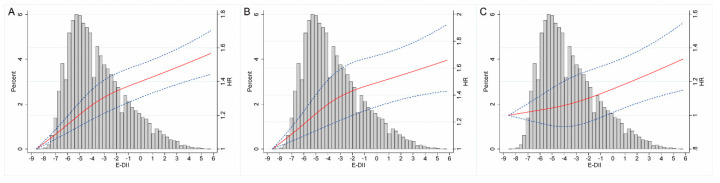
Dose—response analyses for the association between all—cause (**A**), CVD (**B**), or cancer mortality (**C**) and E—DII were performed using a restricted cubic spline model with 3 knots at the 10th, 50th, and 90th percentiles. Red solid lines represent point estimates, and blue dashed lines represent 95% CIs. The histograms show the percentage of participants (left y-axis) belonging to each level of E—DII. CVD—cardiovascular disease; E—DII—Energy—adjusted Dietary Inflammatory Index; CI—confidence interval.

**Figure 2:**
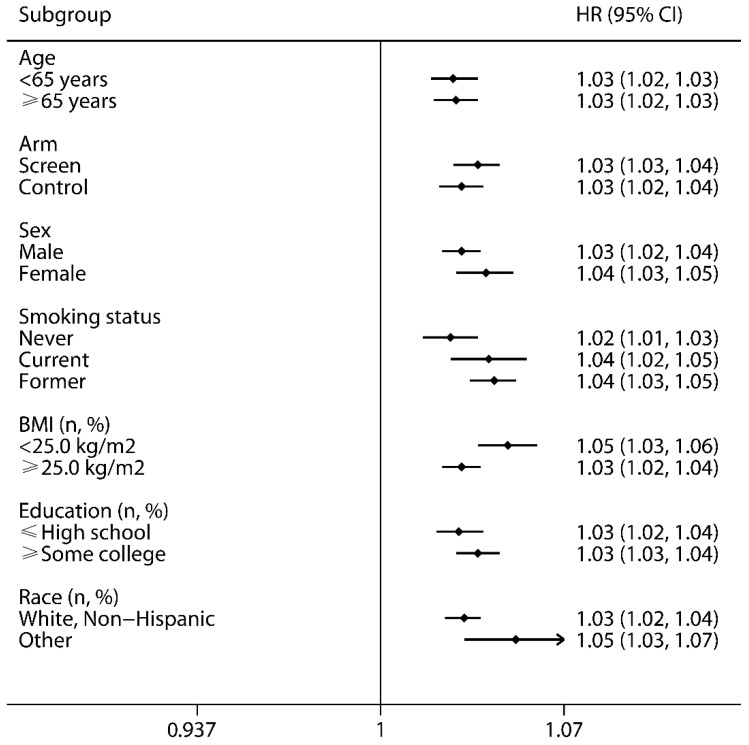
Subgroup analyses were performed based on various potential effect modifiers. The HRs (95% CIs) of one-unit increment in the E-DII were calculated and are shown. E-DII—Energy-adjusted Dietary Inflammatory Index; HRs—hazard ratios; CIs—confidence intervals; BMI—body mass index.

Restricted cubic spline model analysis suggested that there was a nonlinear association of the E-DII with total or CVD mortality (Figure 1A,B, *p* for nonlinearity <0.05). By contrast, the E-DII was linearly related to cancer mortality (Figure 1C, *p* for nonlinearity >0.05). The E-DII remained consistently associated with all-cause mortality in all subgroups, and there was no evidence of an interaction (Figure 2). In sensitivity analysis, the results remained qualitatively similar after excluding events ascertained within 2 or 5 years (data not shown).

## 4. Discussion

In this large prospective US cohort, there was a statistically significant association between the inflammatory potential of the diet, as estimated by the E-DII score, and all-cause mortality. Similar results were obtained for CVD and cancer mortality. The findings from sensitivity analyses and subgroup analyses were consistent with the results of the main analyses.

A positive association of DII with mortality risk among the general population has been indicated in many observational studies, including the Multiethnic Cohort Study [19], the JACC Study [10], the Iowa Women’s Health study [20], and the MONICA/KORA Augsburg Cohort Study [21]. Meta-analyses of cohort studies have also shown that a higher DII was associated with an increased risk of all-cause, CVD, and cancer mortality [11,22,23,24,25,26,27,28,29]. Therefore, most recent reports, including the current analysis, support the association between a pro-inflammatory diet and a higher risk of mortality, although the exact results vary from study to study.

Several dietary metrics have been developed and linked to various health outcomes (e.g., mortality, CVD, type 2 diabetes, and cancer) [30]. Until the DII was created, these dietary metrics belonged to the following three categories: (1) derived from dietary recommendations; (2) related to adherence to a particular dietary cuisine, such as the Mediterranean Dietary Index; or (3) derived from an individual study based on some kind of regression technique [8]. Compared with these approaches, the DII was designed to measure the inflammatory potential by summarizing evidence from a wide variety of human populations using a wide range of methodologies for the study design and dietary assessment. In addition, the DII also collected evidence from reliable laboratory animal and cell culture experiments. The DII has been standardized to dietary intake from representative populations around the world, thus facilitating easy quantitative comparisons across studies [31].

Previous studies have shown a significant association of mortality with various specific food item intakes, including a higher consumption of processed meat [32] and a lower intake of whole grains [33] and fruits and vegetables [34]. A potential limitation of studies that have focused on individual nutrients or food groups is that dietary factors are often inter-correlated, which may result in instability in risk estimation and a possible reduction in statistical power. As a composite of up to 45 food parameters (here 35), the DII obviates problems with these inter-correlations to a large extent.

Several potential mechanisms could explain the association between a pro-inflammatory diet, as shown by higher DII scores, and mortality risk. One possible explanation is that a pro-inflammatory diet will increase the levels of cytokines such as TNF, IL-1, and IFN-g, which may cause the attraction of inflammatory cells into vascular tissue and thus increase the CVD risk [35,36]. Another possible mechanism would be through the effect of a pro-inflammatory diet on insulin resistance by sustaining systemic inflammation [37,38]. Insulin resistance can promote both CVD and cancer [39,40]. Prior studies have found a relationship between higher levels of inflammation and a worse cancer prognosis [41,42].

The major strengths of this study included its prospective cohort design with extended follow-up; a large sample size of participants; detailed information on potential confounders; and the use of a validated DHQ, which covered major parameters that make up the DII. However, as with many studies, some limitations should also be discussed. First, nutritional exposures are often measured with considerable error in commonly used dietary instruments, which may lead to underestimation of the true risk parameter [43]. Errors also may distort risk estimates in other ways, i.e., besides biasing toward the null [44,45]. Nevertheless, the DHQ used in PLCO has been validated against 24-h dietary recalls among a nationally representative sample of 1640 subjects in the Eating at America’s Table Study [15]. Second, over 90% of the subjects analyzed in this study were non-Hispanic Whites; hence, the study results may not be generalizable to other populations. Third, it is possible that the results may be biased by residual or unmeasured confounding even after adjusting for various variables. For example, we could not adjust for physical activity, which has been shown to be linked to mortality risk [46]. Fourth, among the 45 food parameters used for DII construction, only 35 food parameters could be used in the DII calculations in this study, and there may be deviations in the estimation of the possibility of dietary inflammation. Lastly, dietary data were collected at baseline, and thus, the DII was calculated just once. Although the diet tends to be stable in adulthood [47,48], dietary patterns could have changed during the follow-up period.

## 5. Conclusions

In conclusion, the inflammatory potential of the diet, as calculated by the E-DII, was significantly associated with mortality risk in the PLCO cohort. Our findings suggest that diet-related inflammation may increase all-cause and cause-specific mortality in the American population.

## Figures and Tables

**Table 1:** Main characteristics of participants included in this study by E-DII from the Prostate, Lung, Colorectal and Ovarian (PLCO) Screening Trial, 1993 to 2001.

Variables	Q1 (n = 20,368)	Q2 (n = 20,367)	Q3 (n = 20,368)	Q4 (n = 20,367)	Q5 (n = 20,367)	*p*-Value
Age (y), mean (SD)	62.8 (5.3)	62.7 (5.3)	62.4 (5.3)	62.3 (5.3)	61.8 (5.2)	<0.001
Sex (n, %)						
Female	6332 (31.1%)	7705 (37.8%)	9503 (46.7%)	11,642 (57.2%)	14,351 (70.5%)	<0.001
Male	14,036 (68.9%)	12,660 (62.2%)	10,864 (53.3%)	8724 (42.8%)	6015 (29.5%)	
Arm (n, %)						
Screen	10,406 (51.1%)	10,212 (50.1%)	10,419 (51.2%)	10,451 (51.3%)	10,355 (50.8%)	0.140
Control	9962 (48.9%)	10,153 (49.9%)	9948 (48.8%)	9915 (48.7%)	10,011 (49.2%)	
Smoking status (n, %)						
Never	10,590 (52.0%)	10,534 (51.7%)	10,105 (49.6%)	9303 (45.7%)	8064 (39.6%)	<0.001
Current	930 (4.6%)	1254 (6.2%)	1670 (8.2%)	2116 (10.4%)	3442 (16.9%)	
Former	8847 (43.4%)	8574 (42.1%)	8585 (42.2%)	8944 (43.9%)	8854 (43.5%)	
Education (n, %)						
≤High school	6744 (33.1%)	7871 (38.6%)	8463 (41.6%)	9205 (45.2%)	10,684 (52.5%)	<0.001
≥Some college	13,583 (66.7%)	12,443 (61.1%)	11,872 (58.3%)	11,132 (54.7%)	9631 (47.3%)	
BMI (n, %)						
<25.0 kg/m^2^	8800 (43.2%)	7619 (37.4%)	6844 (33.6%)	6046 (29.7%)	5170 (25.4%)	<0.001
≥25.0 kg/m^2^	11,289 (55.4%)	12,494 (61.3%)	13,274 (65.2%)	14,052 (69.0%)	14,895 (73.1%)	
Race (n, %)						
White, Non-Hispanic	18,123 (89.0%)	18,526 (91.0%)	18,691 (91.8%)	18,746 (92.0%)	18,511 (90.9%)	<0.001
Other	2241 (11.0%)	1828 (9.0%)	1670 (8.2%)	1613 (7.9%)	1846 (9.1%)	
Marital status (n, %)						
Married	15,382 (75.5%)	15,706 (77.1%)	15,903 (78.1%)	16,320 (80.1%)	16,367 (80.4%)	<0.001
Not married	4950 (24.3%)	4611 (22.6%)	4430 (21.7%)	4015 (19.7%)	3955 (19.4%)	

E-DII—Energy-adjusted Dietary Inflammatory Index; y—year; SD—standard deviation; BMI—body mass index; Q—quintiles.

**Table 2:** Associations of total mortality, CVD mortality, or cancer mortality with E-DII from the Prostate, Lung, Colorectal and Ovarian (PLCO) Screening Trial, 1993 to 2001.

Variables	Median	Cohort (n)	Cases (n)	Crude HR (95% CI), *p*-Value	Adjusted HR (95% CI) *, *p*-Value
All-cause					
Q1 (<−5.6)	−6.2	20,368	4091	Reference	Reference
Q2 (≥−5.6 to <−4.6)	−5.1	20,367	4512	1.14 (1.09–1.19), *p* < 0.001	1.08 (1.03–1.13), *p* = 0.001
Q3 (≥−4.6 to <−3.4)	−4.0	20,368	4750	1.23 (1.18–1.28), *p* < 0.001	1.12 (1.08–1.17), *p* < 0.001
Q4 (≥−3.4 to <−1.6)	−2.6	20,367	5185	1.37 (1.31–1.42), *p* < 0.001	1.17 (1.12–1.22), *p* < 0.001
Q5 (≥−1.6)	−0.1	20,367	5603	1.54 (1.48–1.60), *p* < 0.001	1.23 (1.18–1.29), *p* < 0.001
				*p* for trend < 0.001	*p* for trend < 0.001
CVD					
Q1 (<−5.6)	−6.2	20,368	1228	Reference	Reference
Q2 (≥−5.6 to <−4.6)	−5.1	20,367	1446	1.22 (1.13–1.31), *p* < 0.001	1.15 (1.06–1.24), *p* = 0.001
Q3 (≥−4.6 to <−3.4)	−4.0	20,368	1472	1.27 (1.18–1.37), *p* < 0.001	1.15 (1.07–1.25), *p* < 0.001
Q4 (≥−3.4 to <−1.6)	−2.6	20,367	1628	1.43 (1.33–1.54), *p* < 0.001	1.22 (1.13–1.32), *p* < 0.001
Q5 (≥−1.6)	−0.1	20,367	1760	1.61 (1.50–1.73), *p* < 0.001	1.30 (1.20–1.41), *p* < 0.001
				*p* for trend < 0.001	*p* for trend < 0.001
Cancer					
Q1 (<−5.6)	−6.2	20,368	1242	Reference	Reference
Q2 (≥−5.6 to <−4.6)	−5.1	20,367	1321	1.09 (1.01–1.18), *p* = 0.026	1.04 (0.96–1.13), *p* = 0.316
Q3 (≥−4.6 to <−3.4)	−4.0	20,368	1379	1.16 (1.08–1.26), *p* < 0.001	1.05 (0.97–1.13), *p* = 0.240
Q4 (≥−3.4 to <−1.6)	−2.6	20,367	1462	1.26 (1.16–1.35), *p* < 0.001	1.04 (0.96–1.12), *p* = 0.355
Q5 (≥−1.6)	−0.1	20,367	1757	1.56 (1.45–1.68), *p* < 0.001	1.14 (1.06–1.24), *p* = 0.001
				*p* for trend < 0.001	*p* for trend = 0.001

CVD, cardiovascular disease; E-DII, Energy-adjusted Dietary Inflammatory Index; HR, hazard ratio; CI, confidence interval; Q, quintiles. * Adjusted for age, sex, race, body mass index, education, smoking status, marital status, randomization arm, aspirin use, history of hypertension, history of diabetes, history of stroke, and history of heart attack.

## Data Availability

The data used in this study can be obtained from the PLCO website (https://cdas.cancer.gov/datasets/plco/ (accessed on 12 February 2020).
